# *In silico* design and validation of a time-varying PID controller for an artificial pancreas with intraperitoneal insulin delivery and glucose sensing

**DOI:** 10.1063/5.0145446

**Published:** 2023-05-23

**Authors:** Alberto Dalla Libera, Chiara Toffanin, Martina Drecogna, Alfonso Galderisi, Gianluigi Pillonetto, Claudio Cobelli

**Affiliations:** 1 Department of Woman and Child's Health, University of Padova, 35128 Padova, Italy; 2 Department of Electrical, Computer and Biomedical Engineering, University of Pavia, 27100 Pavia, Italy; 3 Department of Pediatrics, Pediatric Endocrinology, Yale University, New Haven, Connecticut 06520, USA; 4 Department of Information Engineering, University of Padova, 35131 Padova, Italy

## Abstract

Type 1 diabetes (T1D) is a chronic autoimmune disease featured by the loss of beta cell function and the need for lifetime insulin replacement. Over the recent decade, the use of automated insulin delivery systems (AID) has shifted the paradigm of treatment: the availability of continuous subcutaneous (SC) glucose sensors to guide SC insulin delivery through a control algorithm has allowed, for the first time, to reduce the daily burden of the disease as well as to abate the risk for hypoglycemia. AID use is still limited by individual acceptance, local availability, coverage, and expertise. A major drawback of SC insulin delivery is the need for meal announcement and the peripheral hyperinsulinemia that, over time, contributes to macrovascular complications. Inpatient trials using intraperitoneal (IP) insulin pumps have demonstrated that glycemic control can be improved without meal announcement due to the faster insulin delivery through the peritoneal space. This calls for novel control algorithms able to account for the specificities of IP insulin kinetics. Recently, our group described a two-compartment model of IP insulin kinetics demonstrating that the peritoneal space acts as a virtual compartment and IP insulin delivery is virtually intraportal (intrahepatic), thus closely mimicking the physiology of insulin secretion. The FDA-accepted T1D simulator for SC insulin delivery and sensing has been updated for IP insulin delivery and sensing. Herein, we design and validate—*in silico—*a time-varying proportional integrative derivative controller to guide IP insulin delivery in a fully closed-loop mode without meal announcement.

## I. INTRODUCTION

**FIG. 1.**
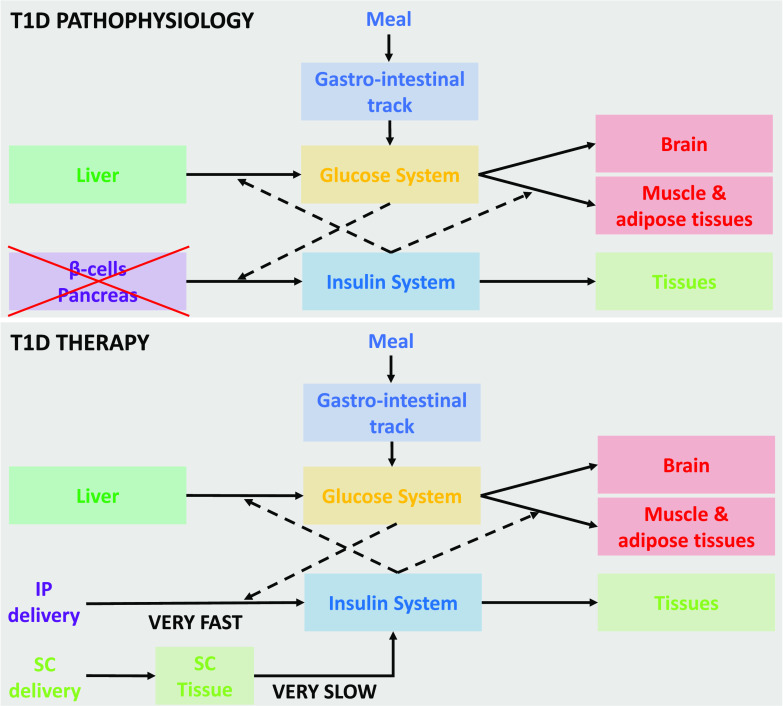
Upper panel: schematic diagram of the T1D pathophysiology; the *β*-cells of the pancreas are not working. Bottom panel: schematic diagram of the T1D therapy; both SC and IP delivery modes are depicted.

Type 1 diabetes (T1D) is a chronic autoimmune disease featured by a progressive loss of beta cell function that requires lifetime insulin replacement (Fig. 1, upper panel). T1D affects ∼1 child out of 200, with a 2% yearly increase in its incidence in youths as a result of a mixture of genetic predisposition and environmental triggers.^1^

Over the recent decade, the use of automated insulin delivery systems (AID)—often referred to as artificial pancreas (AP)—has shifted the paradigm of treatment in both adults and children: the availability of continuous subcutaneous (SC) glucose sensors that guide SC insulin delivery through control algorithms has allowed, for the first time, to dramatically reduce the daily burden of the disease for subjects with T1D and families as well as to abate its major short term complication—the hypoglycemia—and simultaneously reduce the incidence of long-term microvascular complications^2^ (Fig. 1, bottom panel). To date, AID represents the first therapeutic options for pediatric onset T1D,^3^ even though their use is still limited by subjects' acceptance, local availability, coverage, and expertise of the healthcare personnel. Nevertheless, AID does not represent a “cure” and remains a complex instrument that can provide a reasonable improvement of the disease. Additionally, the improvement of glycemic control through SC insulin delivery does not come without a cost: individuals with T1D are exposed to peripheral non-physiologic hyperinsulinemia that, over time, is a major contributor to macrovascular complications, including hearth failure and macrovascular disease that are still three times more frequent in those with T1D respect to their healthy peers. These diseases represent the first cause of mortality even for those who achieve optimal glycemic control over a lifetime.^4^ SC insulin delivery creates a paradoxical peripheral hyperinsulinemia necessary to achieve minimal insulin concentration in the portal system able to inhibit hepatic glucose production, thus, preventing fasting hyperglycemia. This observation has prompted the development of dedicated pumps for intraperitoneal (IP) insulin delivery that provide a more physiological way to optimize glycemic control and prevent peripheral hyperinsulinemia (Fig. 1, bottom panel). Inpatient trials demonstrated that IP insulin delivery can improve glycemic control and spare meal announcement due to the fast insulin delivery route represented by the peritoneum.^5^ This calls for control algorithms able to account for the specificities of IP insulin kinetics. Recently, our group described a two-compartment model of IP insulin kinetics, thus proving that the peritoneal space acts as a virtual compartment, and IP insulin delivery is virtually an intraportal (intrahepatic) delivery, thus mimicking the physiology of insulin secretion.^6^ The FDA-accepted UVA/Padova T1D simulator^7^ for SC insulin delivery and sensing has been updated for IP insulin delivery and sensing. In this paper, we design and validate—*in silico—*a proportional integrative derivative (PID) controller to guide IP insulin delivery in a fully closed-loop mode that does not require meal announcement.

## II. BACKGROUND AND PROBLEM STATEMENT

This section provides the outline of the problem and the system's setup. Finally, we briefly overview related works with particular attention to IP controllers.

### A. Setup description and problem statement

An AP is composed of four main components. The first component is a continuous glucose sensor that measures subcutaneous glucose concentration. The second component—the controller—receives the glucose measurements at a constant rate and communicates to the third component—the insulin pump—which delivers insulin. Finally, a human machine interface (HMI) allows communication between the subject and the AP. For instance, through HMI, subjects can monitor their glucose concentration and announce the meal, including an estimate of its carbohydrate content. The meal announcement is essential in AP with SC sensing and infusion (SC-SC-AP). Indeed, in SC-SC-AP, the controller cannot manage meals just by reacting to the glucose concentration caused by meals due to the slow SC insulin kinetics and to the delay of SC sensing. Generally, the controller implements a feedforward strategy that, based on information coming from meal announcement, computes the insulin bolus. However, meal announcement is a burden for the subject and also bears a considerable risk of hypo- and hyperglycemia, e.g., due to erroneous estimates of the carbohydrate intake.

In this work, we considered an AP with IP glucose sensing and insulin delivery, hereafter referred to as IP-IP-AP. In this setup, in particular, insulin kinetics are much faster than with SC delivery,^8^ and this raises the possibility to keep glucose concentration in the target range without recurring to meal announcement, thus leading to a fully automated AP. In this work, we carried out an *in silico* in-depth feasibility study adopting a PID controller without meal information as a control strategy; then, we propose a novel PID time-varying personalization strategy accounting for intra-day metabolic variability and compare it to the total daily insulin (TDI) dose state-of-the-art strategy. It is worth mentioning that we could have also considered more computationally demanding control strategies such as model predictive control (MPC). However, we focused on PID controllers since IP kinetics is much faster, and PID compared to MPC is simpler and less computational demanding, thus, in an IP-IP-AP, limiting power consumption is extremely important.

### B. Related works

The PID is one of the most popular control algorithms due to its simplicity and effectiveness on a broad class of systems.^9^ The PID controller has only three parameters—*K_p_*, *K_i_*, and *K_d_*—manually adaptable or modifiable in an automatic fashion. Applications of this control strategy proved its remarkable robustness to model mismatch, i.e., discrepancies between the actual system and the model used to adjust the parameters.

For these reasons, several works on SC-SC-AP considered the PID controller as the control strategy,^10,11^ leading also to the realization of a commercial product.^11–13^ In AP applications, the typical procedure implemented to derive the PID parameters requires an average model of the glucose–insulin dynamics. This model, also named a “control-relevant model,”^14^ is a simplified model that encodes relevant characteristics of the glucose–insulin relation. For instance, several works considered linear models.^15^ A standard choice consists of using a third-order linear transfer function (TF) with delay.^14^

These models are used to derive the set of average PID parameters, subsequently tailored to the characteristic of the individual subject. Defining a simple and interpretable personalization strategy of the PID parameters is crucial to improve the usability of AP. A convenient and reasonable personalization strategy consists of adapting the parameters based on simple clinical metrics. For instance, the works in Refs. 11 and 14 proposed a personalization based on the TDI parameter, i.e., the amount of insulin units required by a subject with T1D in a standard day.

However, the slow SC insulin kinetics limit the effectiveness of the PID controller in SC-SC-AP. To avoid hyper-hypoglycemic events, PID controllers are, generally, accompanied by two precautions. The first is the aforementioned meal announcement that turns out to be essential even applying more complex control strategies such as MPC.^16,17^ The second is the insulin feedback (IFB),^11^ a feedback on the plasma insulin concentration that limits insulin infusion if plasma insulin concentration becomes too elevated. It is worth noting that the plasma insulin concentration cannot be measured in real-time, so the controller uses an estimate of the plasma insulin, which requires the characterization of a subject-dependent model.

A promising solution to avoid peripheral hyper-insulinemia and optimize post-prandial glucose control without meal announcement is the IP-IP-AP. The pilot study presented in Ref. 18 demonstrated the great potential of IP infusion. In that study, ten patients participated to an inpatient study wearing a SC sensor and an IP infusion pump, controlled by a Zone MPC algorithm. The 24-h clinical protocol included three unannounced meals. The study showed that AP with IP infusion can achieve significantly better glucose control compared to SC infusion. Recent advances in the realization of this technology calls for the derivation of IP glucose–insulin models,^6,19,20^ new tools for *in silico* experiments, and increased attention in the study of control algorithms for IP-IP-AP. An *in silico* preliminary study was presented in Ref. 21, where the authors designed a PID controller for a fully implantable IP-IP-AP on an early version of the UVA/Padova simulator (T1DS) without inter- and intra-day variability, equipped with a population of only ten virtual patients. Like other PID controllers for SC-SC-AP, the algorithm personalizes the PID parameters through the TDI. Other *in silico* studies with MPC were conducted in Refs. 17 and 20. With respect to the modeling, in Ref. 19, the authors derived an animal model of glucose–insulin dynamics, while the work in Ref. 6 derived a two-compartment model of IP insulin kinetics in humans.

## III. PROPOSED APPROACH

This section describes the derivation of the proposed PID controller. We carried out *in silico* experiments on the latest version of the T1DS modified to describe sensing and infusion through the IP route (IP-T1DS), as detailed in Sec. VI A. The simulator is equipped with a virtual population of 100 adult patients,^7^ also describing inter- and -intra-day variability of insulin sensitivity.^22^

Similarly to previous PID approaches,^11,21^ we implemented the three following steps to derive our PID controller:

• identification of a control-relevant model for IP-IP-AP;
• derivation of the average PID parameters;
• implementation of a personalization strategy based on clinical parameters.

In the following, we detail how the three steps have been implemented.

### A. Identification of a control-relevant model for IP-IP-AP

Following the same approach implemented by previous works for SC-SC-AP, as a first step, we derived a control-relevant model of the insulin–glucose relation. These kinds of models do not aim at describing precisely the insulin–glucose TF as models derived for simulation purposed relying on properly collected data, see, for instance, Refs. 7 and 22. To obtain an accurate description of the insulin–glucose TF, we should rely on complex and highly personalized models that, generally, are hard to be used in a principled way to derive a controller. Instead, control relevant models have to provide a simple and compact description of the insulin–glucose TF, useful to derive first-attempt parameters. For this reason, we modeled the insulin–glucose TF with a linear and time-invariant model, and we averaged the results over all the virtual patients.

We identified the glucose–insulin TF starting from data collected by simulating a 24-h protocol without meals in the 100 *in silico* patients of the IP-T1DS. In this way, the glucose dynamic depends only on endogenous glucose production and infused insulin. For each virtual patient, the insulin signal adopted to excite the system is a distinct realization of a zero mean Gaussian noise, filtered with a low pass filter. The cutoff frequency of the low pass filter is $1/30\;({\text{min}}^{-1})$, which guarantees a sufficient excitation in the frequency range of interest. Negative values were saturated to zero. In this experiment, we set the standard deviation of the measurement noise to zero.

**FIG. 2.**
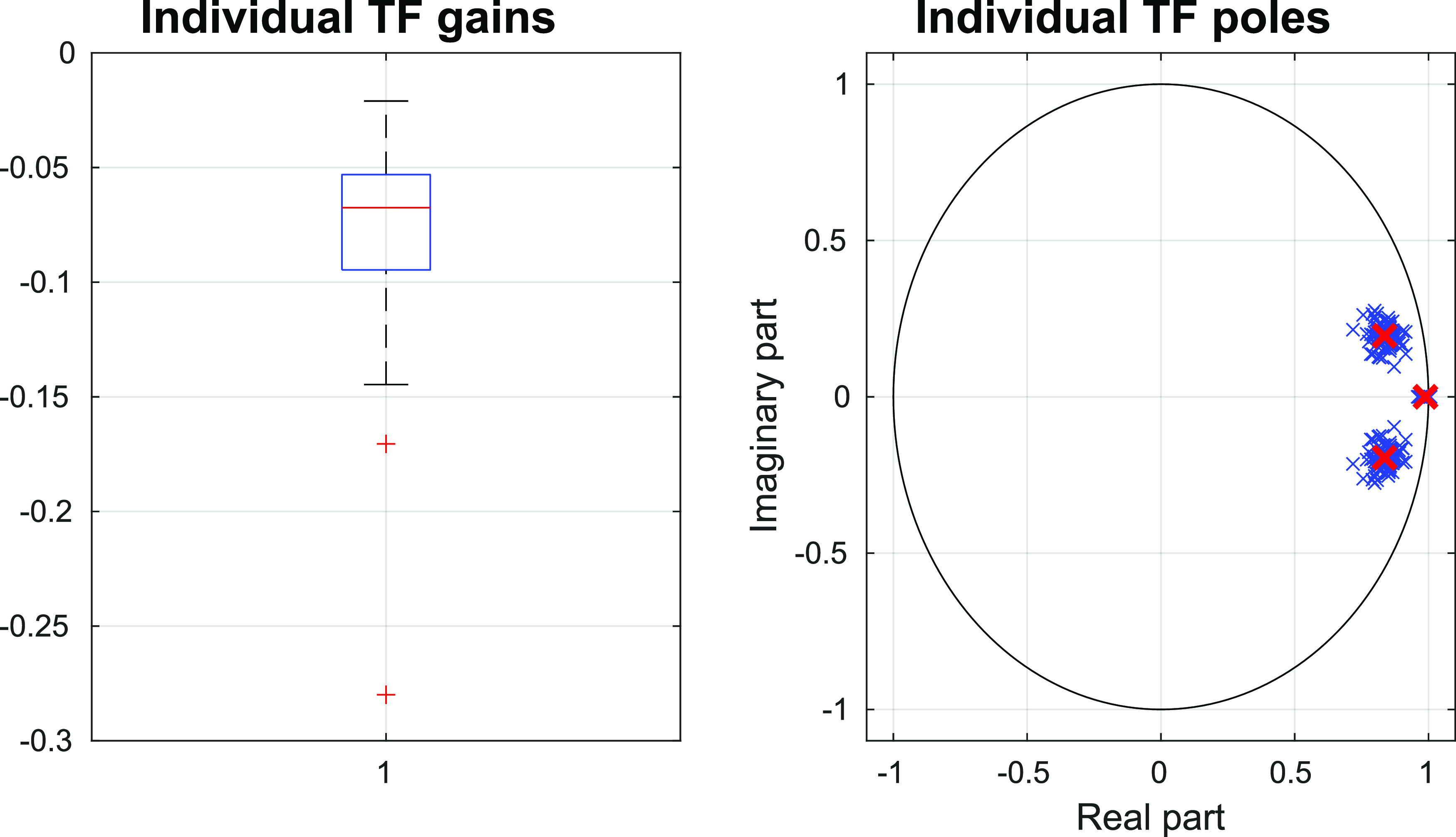
Distributions of the 100 TF gain (left) and 300 poles (right).

We modeled the input–output relation with a third-order ARX model with delay. Let *I*(*k*) and ${\hat{G}}_{m}(k)$ be, respectively, the insulin input rate [U/h] and the measured blood glucose concentration (mg/dl) at time step *k*, eventually filtered using previous measurements. We assumed the following model:

$$\begin{array}{c} {\hat{G}}_{m}(k)+a_{1}{\hat{G}}_{m}(k-1)+a_{2}{\hat{G}}_{m}(k-2)+a_{3}{\hat{G}}_{m}(k-3)\\ =bI(k-3)+e(k)+c_{e}, \end{array}$$ (1)

where *b*, *a*_1_, *a*_2_, and *a*_3_ are the ARX coefficients, *c_e_* is a constant that accounts for endogenous production, and *e*(*k*) is the Gaussian noise. As detailed in Sec. VI B, for each patient, we identified the ARX coefficients, and we derived the correspondent glucose–insulin TF, described by the following equation:

$$\frac{{\hat{G}}_{m}(z^{-1})}{I(z^{-1})}=\frac{Kz^{-3}}{\left(1-p_{1}z^{-1})(1-p_{2}z^{-1})(1-p_{3}z^{-1}\right)},$$

where $z^{-1}$ is the backward shift operator, while *K* and *p_i_* with *i* = 1, 2, and 3 are, respectively, the TF gain and poles. With regard to the distribution of the TF poles, each patient has a real pole with module close to 1 and pair of complex-conjugate poles with faster dynamics. (On average, the real part is 0.8353.) Figure 2 reports the distribution of the individual poles and a boxplot of the individual TF gains. We computed the poles and gain of the average TF by averaging the collected results, thus obtaining

$$\begin{array}{cc} & {\hat{p}}_{1}=0.9887,\\ & {\hat{p}}_{2}=0.8353+0.1949i.\\ & {\hat{p}}_{3}=0.8353+0.1949i,\\ & \hat{K}=-0.0769. \end{array}$$ (2)

Then, the expression of the average TF is

$$\frac{{\hat{G}}_{m}(z^{-1})}{I(z^{-1})}=\frac{-0.0769z^{-3}}{\left(1-0.9887z^{-1})(1-1.6706z^{-1}-0.7357z^{-2}\right)}.$$ (3)

### B. Derivation of the average PID parameters

We derived the average PID parameters based on the properties of the step response obtained on the average glucose–insulin TF in Eq. (3). Let *R*(*k*) and $E(k)=R(k)-{\hat{G}}_{m}(k)$ be, respectively, the reference signal of the PID controller and the tracking error. As reference, we considered a constant signal centered in the middle of the tight euglycemic range, i.e., 110 (mg/dl). The following expression gives the insulin infusion rate of the controller:

$$I(k)=K_{p}E(k)+K_{d}\dot{E}(k)+K_{i}\int_{0}^{k}E,$$ (4)

where *K_p_*, *K_d_*, and *K_i_* are the PID parameters, while $\dot{E}(k)$ and $\int_{0}^{k}E$ are, respectively, the error derivative and integral.

As regards the computation of tracking error's derivative, we considered the following approximation:

$$\dot{E}(k)=\frac{E(k)-E(k-1)}{T},$$

where T=5 (min) is the control period. We remark that the above expression is effective only when the noise corrupting the glucose signal provided to the controller is not too high. In our setup, glucose measurement is filtered onboard by the glucose sensor at a higher frequency, as described in Sec. VI, leading to sufficiently stable estimates.

A crucial aspect of PID controllers for AP is the computation of the tracking error's integral, i.e., $\int_{0}^{k}E$. Indeed, during meals, the tracking error is high and, if not properly managed, the integral contribution assumes high values for an extended interval (known as windup effect), leading to possible hypo-glycemic events. Typical strategies developed to face this issue involve saturation of the integral and attenuation of the terms integrated when the error module is large, see, for instance, the anti-reset windup strategy adopted in Refs. 11 and 21. The integration update rule implemented by our controller is

$$\int_{0}^{k}E=\{\begin{array}{ll} \int_{0}^{k-1}E+TE(k) & \text{if}\;{\hat{G}}_{m}(k)\leq 140\;(\text{mg}/\text{dl}),\\ \int_{0}^{k-1}E & \text{if}\;{\hat{G}}_{m}(k)>140\;(\text{mg}/\text{dl}). \end{array}$$

The algorithm updates the integral only if the glucose is below the upper bound of the tight euglycemic range [80–140] (mg/dl), i.e., the error integrated is set to zero if ${\hat{G}}_{m}(k)>140\;(\text{mg}/\text{dl})$. This rule is motivated by the following rationale. The integral action can compensate disturbances with null or almost null frequency such as the endogenous glucose production. Ideally, at equilibrium, the integral contribution should estimate the basal rate necessary to compensate endogenous production. In contrast, meals are high-frequency disturbances that the integral action cannot compensate. When ${\hat{G}}_{m}(k)>140\;(\text{mg}/\text{dl})$, with high probability, the patient is in a prandial or post-prandial phase. In these configurations, the tracking error is mostly due to meal effects, and it is not representative of the difference between the integral action and the ideal basal rate. So, when ${\hat{G}}_{m}(k)>140\;(\text{mg}/\text{dl})$, we keep the integral constant.

We remark that, in our controller, we do not rely on IFB, since as showed by results in Sec. IV, in the IP-IP-AP we can derive effective controllers also without relying on IFB. As briefly discussed before, the plasma insulin concentration cannot be measured in real-time, and they must be estimated through a model, eventually subject-dependent to obtain accurate estimates. Generally, patients do not have this kind of characterization among their clinical parameters.

We tuned the PID parameters based on the properties of the step response obtained on Eq. (3). The result of the tuning process is a trade-off between raising time and overshoot performance. The raising time is the time the response takes to rise from 10% to 90% of the final step value. Instead, the overshoot is the maximum value exceeding the final step value expressed as a percentage of the final step value. In our application, a small rising time denotes a prompt response of the controller to meals, while high overshoot values could lead to hypo-glycemic events. Ideally, we would like to obtain a fast response by keeping the overshoot limited. However, typically, too small rising times are accompanied by a considerable overshoot. The raising time and the overshoot obtained after tuning are, respectively, 130 (min) and 2.33%. The values of the PID parameters are

$$\begin{array}{cc} & K_{p}=-0.0665,\\ & K_{i}=-1.9342\times 10^{-4},\\ & K_{d}=-2.0922. \end{array}$$ (5)

### C. PID time-varying personalization

The PID parameters in Eq. (5) do not account for subject-variability. Generally, the control parameters are tailored to the characteristic of the single patient based on simple clinical parameters, like the TDI. From a control point of view, the idea is to assume that the patients transfer functions have the same poles, but different gains. The gain of each patient is the average TF gain multiplied by a coefficient K′ that depends on clinical parameters. Then, for each patient, the correspondent PID parameters are equal to the average PID parameters in Eq. (5) scaled by 1/K′. For instance, when TDI is considered for personalization, K′ is inversely proportional to it. Indeed, patients with low TDI are insulin-sensitive; consequently, the gain of their glucose–insulin TF is higher than the one of patients with high TDI.

The TDI-based personalization does not account for intra-day variability since the TDI provides a global description of the patient's insulin sensitivity. Insulin sensitivity can vary during the day. For instance, a model of the insulin sensitivity variation was presented in Ref. 22. This model splits the day into three parts, breakfast, lunch, and dinner, and assigns a different insulin sensitivity to each part of the day.

**FIG. 3.**
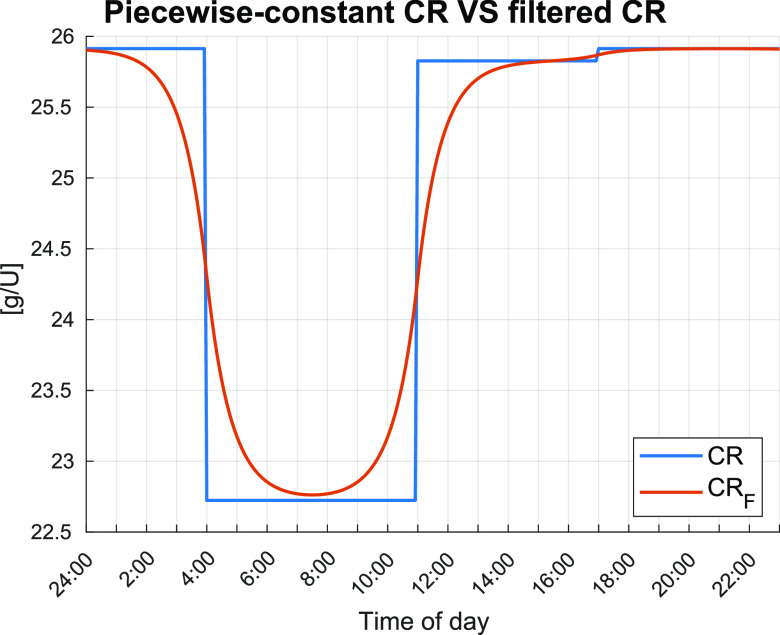
Example of the piecewise-constant CR and filtered CR as a function of time of day.

To account for intra-day variability, we considered a different parameter for personalization. A clinical parameter closely related to insulin sensitivity is the carbohydrate to insulin ratio (CR). Patients use the CR to compute the insulin bolus necessary to compensate a given meal. The insulin bolus is computed by dividing the amount of meal carbohydrates by the CR. Then, CR is proportional to insulin sensitivity. Typically, each patient has three values of CR, associated with breakfast, lunch, and dinner, hereafter denoted *CR_B_*, *CR_L_*, and *CR_D_*. We defined K′ as a function of *CR_B_*, *CR_L_*, and *CR_D_* and the time of the day *h*. Drastic variations of the PID parameters could lead to instabilities, besides not being physiologically plausible. In our algorithm, to avoid discontinuities of the PID parameters, K′ is a function of $CR_{F}(h)$, a smoothed version of the piecewise-constant signal defined by *CR_B_*, *CR_L_*, and *CR_D_*. Specifically, we obtained $CR_{F}(h)$ by filtering the piecewise-constant signal with an acausal low-pass filter with cutoff frequency of $1/300\;({\text{min}}^{-1})$. Figure 3 compares the evolution of the piecewise-constant signal and its filtered version as a function of the time of the day for one of the 100 virtual patients.

The expression of the time-varying personalization gain K′(h) is

$$K\prime (h)=\frac{CR_{F}(h)}{CR_{M}},$$ (6)

where $CR_{M}=39.8381[g/U]$ is the maximum CR over all the 100 virtual patients. Of note CRM is the maximum of the CR distribution, very unlikely to occur. The division by *CR_M_* is needed to normalize $CR_{F}(h)$. Then, in our algorithm, the time-varying personalized PID parameters at time *h* are given in the following expression:

$$\begin{array}{cc} & K_{p}(h)=-0.0665/K\prime (h),\\ & K_{i}(h)=-1.9342\times 10^{-4}/K\prime (h),\\ & K_{d}(h)=-2.0922/K\prime (h). \end{array}$$ (7)

## IV. EXPERIMENTS

We tested the effectiveness of the IP-IP-AP without meal announcement through extensive *in silico* experiments by simulating a 12-weeks protocol. The protocol includes three meals per day, at variable times and with variable amounts. Each day, the meals time and CHO amount were sampled from uniform distributions with the following ranges:

• Breakfast: time [7:00–8:30], CHO [40 ± 20%].
• Lunch: time [12:30–13:30], CHO [80 ± 20%].
• Dinner: time [19:30–20:30], CHO [60 ± 20%].

Additionally, to further increase variability, we included in the protocol two random snacks, one after breakfast and one after lunch. Each snack can occur with probability 50% approximately two hours after breakfast or lunch. The amount of carbohydrates per snack is sampled from a uniform distribution with ranges [20 ± 20%] CHO.

To evaluate the performance of our time-varying personalization, we also implemented a personalization based on TDI as a baseline. Due to the modifications of the simulator (see Sec. VI A), the direct application of previous TDI-based approaches did not prove effective, so we implemented our TDI-based personalization following the same procedure considered for CR. Specifically, we defined the personalization coefficient K′ as follows:

$$K\prime =\frac{TDI_{M}}{\mathrm{TDI}},$$

where $TDI_{M}=97.9932[U]$ is the maximum value of the virtual patients' TDIs. The personalized control parameters are defined as in Eq. (7).

The simulator considered is a modified version of the UVA/Padova T1DS equipped with the FDA-accepted S2017 population.^7^ Section VI A briefly describes the modifications implemented to account for IP sensing and infusion, as well as variations of insulin sensitivity. In this work, we considered two setups, referred to as a standard setup and a robust setup. The first setup considers random deviations of the insulin sensitivity in the range ±40% around the nominal values reached at breakfast, lunch, and dinner, while the second also considers an additional random shift of ±30%, to further stress robustness with Respect to deviations from the nominal values. A detailed description of the setup is reported in Sec. VI A.

For each personalization, we computed different standard metrics^23^ detailed in Sec. VI C. In Table I, we reported the statistics of each metric for the following parts of the day:

•
D&N: day and night;
• *PP*: post-prandial period (4 h after meals).

**TABLE I.** Glucose metrics of the 12-week simulation in the standard setup. Statistically relevant results are highlighted in bold.

		D&N	***PP***
***GM***	TDI	116.38 [113.19, 118.89]	122.12 [117.75, 126.54]
	CR	115.77 [113.55, 117.74]	120.05 [117.89, 123.20] ^a^
***GSTD***	TDI	18.12 [13.94, 22.43]	19.52 [14.46, 24.56]
	CR	15.37 [12.29, 19.24] ^b^	16.48 [13.26, 22.15] ^b^
HT%	TDI	**13**	9
	CR	2 ^c^	2
HT#	TDI	0 [0, 0]	0 [0, 0]
	CR	0 [0, 0]	0 [0, 0]
***TR***	TDI	99.71 [97.85, 100]	99.54 [96.47, 100]
	CR	99.94 [98.84, 100]	99.90 [98.04, 100]
***TTR***	TDI	88.87 [83.85, 94.35]	81.67 [73.64, 90.75]
	CR	92.47 [88.45, 95.50] ^b^	87.59 [81.30, 92.58] ^b^
TA180	TDI	0 [0, 2]	0 [0, 3]
	CR	0 [0, 1]	0 [0, 2]
TA250	TDI	0 [0, 0]	0 [0, 0]
	CR	0 [0, 0]	0 [0, 0]
TB70	TDI	0 [0, 0]	0 [0, 0]
	CR	0 [0, 0]	0 [0, 0]
***LBGI***	TDI	0.22 [0.13, 0.33]	0.15 [0.07, 0.24]
	CR	0.14 [0.08, 0.22] ^b^	0.09 [0.04, 0.15] ^b^
***HBGI***	TDI	0.57 [0.31, 0.87]	0.91 [0.47, 1.37]
	CR	0.41 [0.25, 0.62] ^b^	0.64 [0.39, 1.00] ^b^

^a^ Statistical significance level: p-value < 0.01.

^b^ Statistical significance level: p-value < 0.001.

^c^ Statistical significance level: p-value < 0.05.

Statistically significant results are highlighted in bold (see Sec. VI C for more details on metrics and statistical tests).

Results show that both controllers achieve remarkable glycemic control performance. The proposed time-varying CR-based PID outperforms the TDI-based PID regarding the time percentage spent in the tight euglycemic range (TTR). In addition to that, the personalization through CR reduces the risk of hypoglycemic events: with TDI-based PID 13% of the patients needed at least one hypo-treatment, while with the one based on CR only 2% of the patients were involved. Moreover, with the CR-based PID the glucose standard deviation (GSTD) is significantly lower. This fact suggests that by modulating the PID parameters during the day, the controller fits better to variations of insulin sensitivity.

**FIG. 4.**
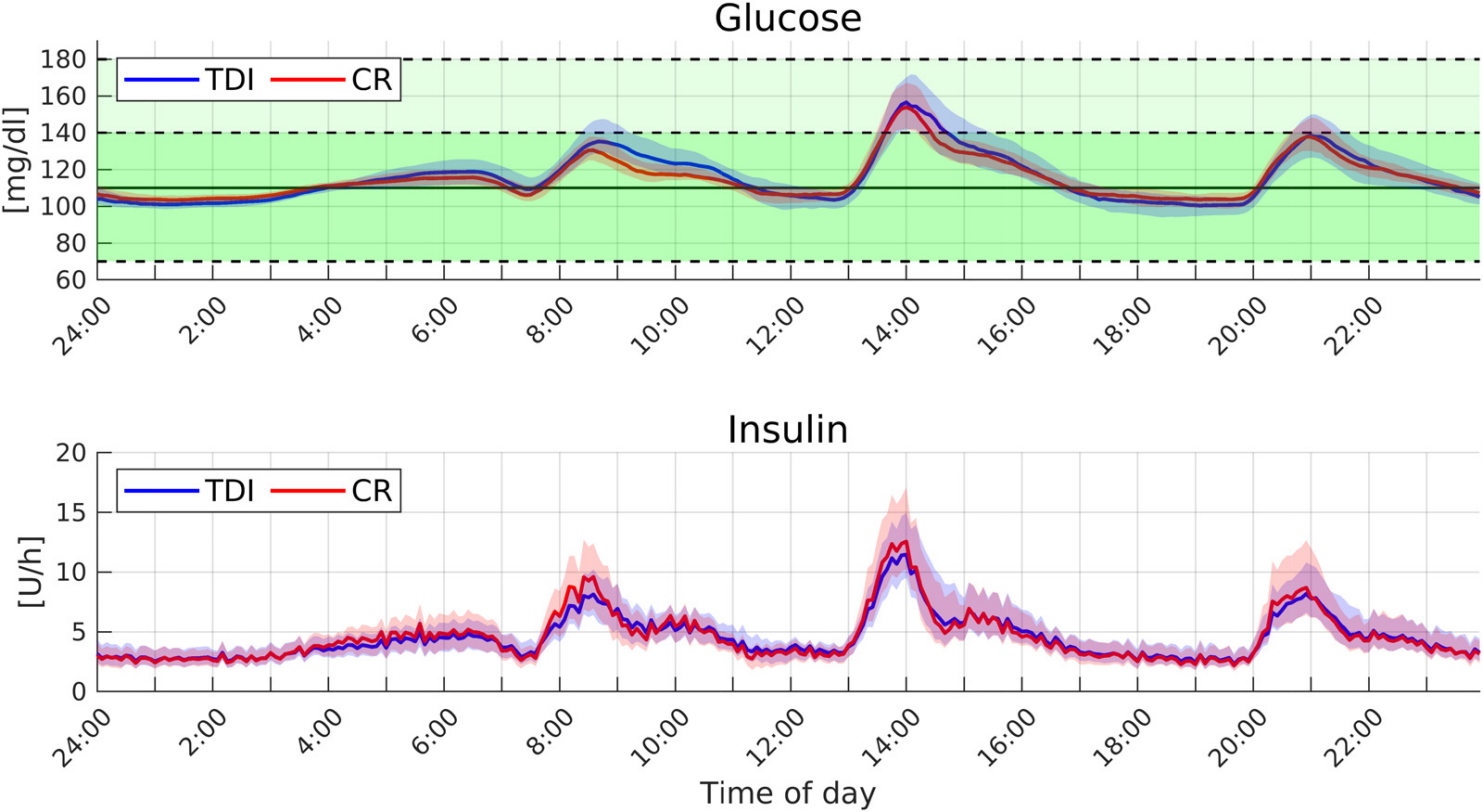
Average time evolution of the glucose and insulin (median and confidence intervals) obtained with the PID controller based on TDI and CR personalizations in the standard setup.

Similar qualitative considerations can be done by analyzing Fig. 4, where we reported the median and the first and third quartiles of the average glucose–insulin time course. For each patient, we computed the average day-glucose-insulin evolution over the 12 weeks; then we reported the median and the first and third quartiles among the 100 patients. While the two controllers perform similarly at lunch and dinner, the CR-based PID performs better at breakfast. The improvement is probably related to the ability of the CR-based PID to modify its gains to compensate for insulin resistance that affects several patients in the morning, while TDI-based personalization keeps the parameters unchanged.

**FIG. 5.**
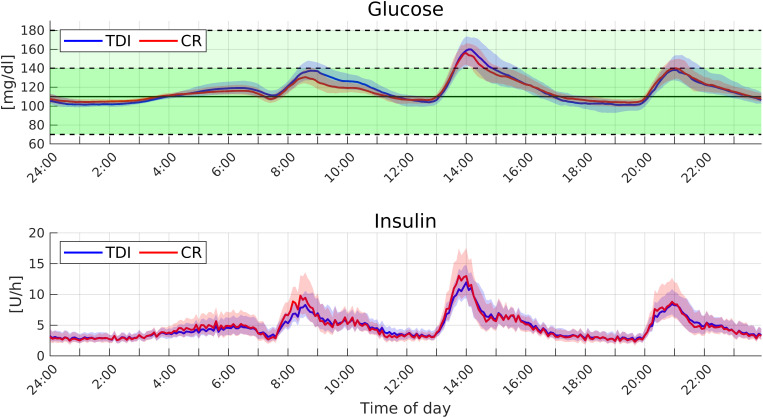
Average time evolution of the glucose and insulin (median and confidence intervals) obtained with the PID controller based on TDI and CR personalizations in the robust setup.

Figure 5 reports the median and the first and third quartiles of the average glucose-insulin time course collected in the robust setup, while the statistics of performance metric are in Table II. The controllers performed similar to the nominal setup, proving robustness with respective to considerable variations of the insulin sensitivity. Also in this more challenging setup, the time-varying PID outperforms the standard TDI based controller in terms of TTR.

**TABLE II.** Glucose metrics of the 12-week simulation in the robust setup. Statistically relevant results are highlighted in bold.

		D&N	***PP***
***GM***	TDI	116.34 [113.87, 121.82]	122.07 [117.14, 130.38]
	CR	113.69 [113.64, 119.89]	121.94 [117.12, 126.61] ^a^
***GSTD***	TDI	18.95 [15.58, 22.30]	20.55 [15.34, 23.97]
	CR	15.91 [12.62, 20.24] ^b^	17.64 [13.60, 22.45] ^b^
HT%	TDI	**12**	10
	CR	7	6
HT#	TDI	0 [0, 0]	0 [0, 0]
	CR	0 [0, 0]	0 [0, 0]
***TR***	TDI	99.44 [97.49, 100]	99.08 [96.02, 100]
	CR	99 [98, 100] ^c^	99.75 [97.19, 100]
***TTR***	TDI	87.36 [80.46, 94.01]	80.14 [68.90, 90.13]
	CR	91.11 [85.52, 94.78] ^b^	85.41 [76.15, 91.40] ^b^
TA180	TDI	0.44 [0, 2.19]	0.73 [0, 3.62]
	CR	0.12 [0, 1.70]	0.20 [0, 2.80]
TA250	TDI	0 [0, 0]	0 [0, 0]
	CR	0 [0, 0]	0 [0, 0]
TB70	TDI	0 [0, 0]	0 [0, 0]
	CR	0 [0, 0]	0 [0, 0]
***LBGI***	TDI	0.19 [0.11, 0.32]	0.12 [0.05, 0.23]
	CR	0.12 [0.06, 0.20] ^b^	0.07 [0.03, .14] ^a^
***HBGI***	TDI	0.64 [0.34, 0.94]	0.95 [0.46, 1.55]
	CR	0.47 [0.28, 0.77] ^b^	0.76 [0.43, 1.15] ^b^

^a^ Statistical significance level: p-value < 0.01.

^b^ Statistical significance level: p-value < 0.001

^c^ Statistical significance level: p-value < 0.05.

## V. CONCLUSIONS

In summary, we have designed and validated *in silico* a time-varying PID controller for a fully implantable intraperitoneal AP, without meal announcement. We also implemented a standard time-invariant PID controller personalized through TDI, and we compared the two control strategies through extensive in-silico experiments. Compared to previous in-silico studies, we used the FDA-accepted T1D simulator, equipped with a richer virtual population of 100 patients and inter- and -intra-day variability, properly modified to account for IP delivery. Collected results suggest that a PID controller can be effective in IP-IP-AP applications, even without relying on meal announcement. Furthermore, time-varying personalization based on circadian variations of insulin sensitivity increases glucose control performance compared to a time-invariant individualization based on subject's total daily insulin. The absence of meal announcement is a major advantage of an IP fully closed-loop system.

In our future works, besides considering *in vivo* tests, we will study the possibility of adapting the PID parameters based on data collected on the system, for instance, relying on adaptive control strategies, run-to-run approaches,^24^ or data-driven methods^25^

## VI. METHODS

### A. Simulator

The simulator is equipped with the FDA-approved S2017 population^7^ composed of 100 virtual patients with inter- and -intra-day variability of insulin sensitivity.^22^ In our experiments, we considered two setups, hereafter referred to as the standard setup and the robust setup. The standard setup corresponds to the setup described in Ref. 22. Each virtual patient has a nominal insulin sensitivity daily path that connects smoothly the values of insulin sensitivity at breakfast, lunch, and dinner. To account for inter-day variation of insulin sensitivity, every day the nominal path is perturbed by multiplying the sensitivity values reached at breakfast, lunch, and dinner by a Gaussian noise with mean 1 and standard deviation 0.2, so that variations in the range ±40% occur with probability 95.45%. In the second setup—the robust setup—the insulin sensitivity of each patient varies following the same rules of the standard setup, but it is shifted randomly of ±30%. In this way, the robust setup tests the robustness of controller with respective to additional systematic errors in the nominal clinical parameters—CR and TDI—provided by the simulator. In addition, in both the setups, the simulator models also the so-called “dawn” phenomenon, that is, the increase in the blood glucose concentration that occurs during early morning hours, due to an increased endogenous glucose production.

**FIG. 6.**
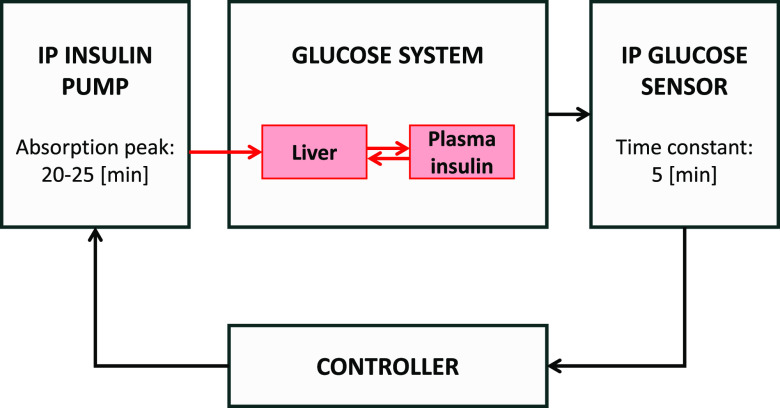
Schematic representation of the IP-IP AP simulator. The insulin subsystem is evidenced in red.

**FIG. 7.**
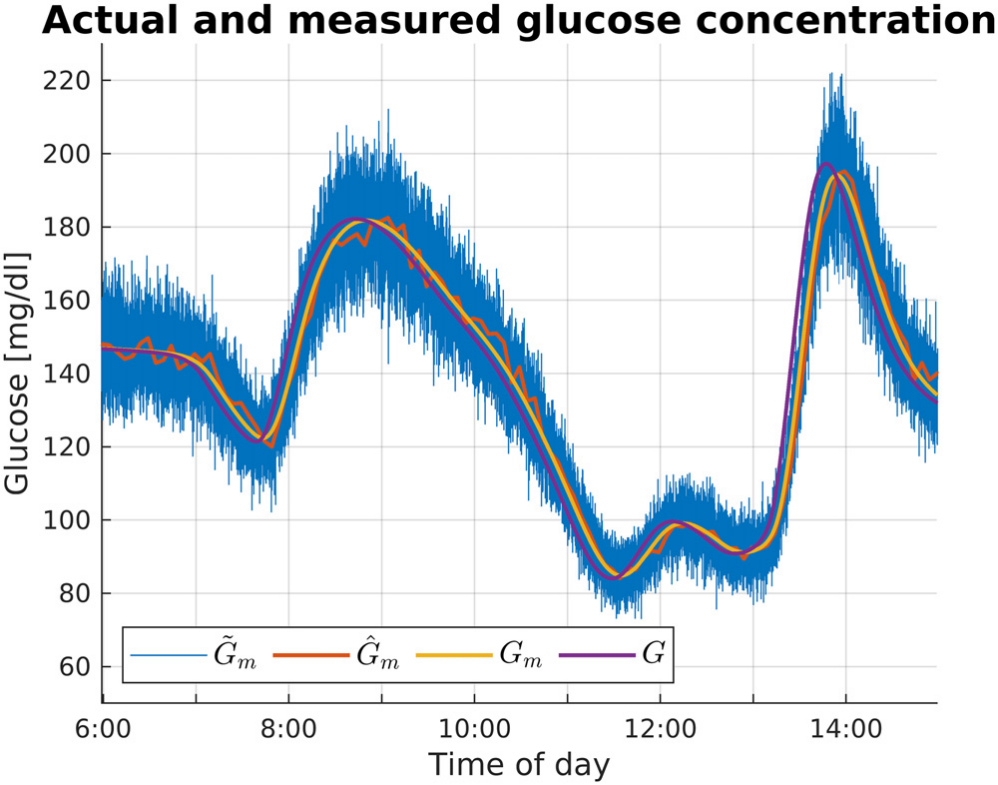
Example of the actual, measured, and filtered glucose concentration time course.

As outlined in Fig. 6, we modified the simulator to account for IP infusion and sensing. Specifically, as demonstrated in Ref. 6, we approximated IP infusion by delivering insulin in the liver compartment instead of the subcutaneous tissue. As regards the sensing system, we simulated the model identified in Ref. 8, described by the following equation:

$$\frac{dG_{m}(t)}{dt}=\frac{1}{\tau }\left(G(t-\theta )-G_{m}(t)\right),$$

where *G*(*t*) and $G_{m}(t)$ are, respectively, the blood glucose and the measured glucose at time *t*, while *θ* and *τ* are, respectively, the time delay and the model time constant. We assumed the average values reported in Ref. 8, i.e., θ=0,68 (min) and τ=5.6 (min). In addition, measurements were corrupted by zero-mean Gaussian noise, with a standard deviation equal to the 5% of the current glucose level. The sensor implements filtering operations onboard through a moving average filter that returns the average of the last ten samples collected with period 30 (s). The controller receives a filtered measure at constant rate with period 5 (min). Figure 7 reports an example of the time course of *G*, *G_m_*, ${\tilde{G}}_{m}$, and ${\hat{G}}_{m}$, where ${\tilde{G}}_{m}$ and ${\hat{G}}_{m}$ are, respectively, *G_m_* corrupted by the Gaussian noise and the output of the moving average filter.

### B. ARX model identification

For each of the 100 virtual patients, we identified an individual ARX model of the kind in Eq. (1) based on data collected with the 24-h meals-free protocol described in Sec. III A. We estimated the ARX model coefficients by solving a linear least squares. We arranged the ARX coefficients in a vector *α*, namely,

$$\alpha ={\left[\begin{array}{ccccc} a_{1} & a_{2} & a_{3} & b & c_{e} \end{array}\right]}^{T}.$$

The output vector *Y* and the regression matrix *X* of the least squares problem are

$$\begin{array}{c} Y={\left[\begin{array}{ccc} G_{m}(k_{1}) & \cdots & G_{m}(k_{N}) \end{array}\right]}^{T},\\ X=\left[\begin{array}{ccccc} -G_{m}(k_{1}-1) & -G_{m}(k_{1}-2) & -G_{m}(k_{1}-3) & I(k_{1}-3) & 1\\ \vdots & \vdots & \vdots & \vdots & \vdots \\ -G_{m}(k_{N}-1) & -G_{m}(k_{N}-2) & -G_{m}(k_{N}-3) & I(k_{N}-3) & 1 \end{array}\right], \end{array}$$

where $k_{1}\ldots k_{N}$ denotes the time instants at which date were collected. Then, the least squares estimates of *α* are

$$\hat{\alpha }={\left[\begin{array}{ccccc} {\hat{a}}_{1} & {\hat{a}}_{2} & {\hat{a}}_{3} & \hat{b} & {\hat{c}}_{e} \end{array}\right]}^{T}={\left(X^{T}X\right)}^{-1}X^{T}Y.$$

Finally, starting from $\hat{\alpha }$, we computed the individual TF, obtaining the poles and gains reported in Fig. 2.

### C. Metrics and statistical tests

To compare the performance of the two personalization strategies, we computed different standard metrics.^23^

Table I reports the statistics of the following metrics:

• GM: glucose mean;
• GSTD: glucose standard deviation;
• HT%: percentage of patients that needed at least 1 hypo treatment in closed-loop;
• HT#: number of hypo treatments;
• TR: time percentage with *G* in the range [70–180];
• TTR: time percentage with *G* in the range [70–140];
• TA180: time percentage with *G* > 180;
• TA250: time percentage *G* > 250;
• TB70: time *G* < 70;
• LBGI: low blood glucose index;
• HBGI: high blood glucose index.

For all the continuous variables, we reported the median and the confidence intervals ( 25–75%). Statistically significant results are highlighted in bold. We performed two kinds of statistical tests: (i) Mann–Whitney U test for continuous variables, using the MATLAB function test; (ii) Fisher's exact test for categorical variables ( HT%), using the MATLAB function fishertest.

## Data Availability

The data that support the findings of this study are available from the corresponding author upon reasonable request.
